# Nitrous Oxide Gas as an Anœsthetic

**Published:** 1866-04

**Authors:** 


*Nitrous Oxide Gas as an Anœsthetic.*—In the *Medical and Surgical Reporter*, Dr. J. W. Carnochan, Surgeon-in-Chief to the State Emigrant's Hospital, New York, reports three surgical operations which he performed, using the nitrous oxide gas as the anæsthetic. The first operation was for the removal of the entire breast and glands of the axilla for cancer. The patient, a lady in feeble health, was kept in a profound anæsthetic sleep for sixteen consecutive minutes. No nausea or vomiting followed the administration.

The second was an amputation of the leg. The patient, an adult extremely debilitated, was kept under the influence of the anæsthetic for two minutes and a quarter. No nausea or any unpleasant symptoms followed. The third, an amputation of the leg in a boy thirteen years of age; the whole time consumed in the inhalation, operation, and recovery from the anæsthetic sleep was only two minutes. No unpleasant symptoms followed. After the report of these cases—a synopsis of which we have given above—Dr. Carnochan, in discussing the advantages of this agent, says:

"For minor operations, or for capital operations, such as amputations which, when properly performed, should require but a few minutes, I have no hesitation in stating that the nitrous oxide gas, as an anæsthetic is far superior to either chloroform or ether. Insensibility is suddenly produced, and the patient recovers consciousness quickly, the operation being attended by no nausea or sickness, and without the dangerous effects often incident to chloroform and ether.

" It is worthy of remark that the nitrous oxide gas approximates in its chemical combination to the composition of the ordinary atmosphere, and we may thus, inferentially, account for its favorable influence. Whether it can be used in operations which, from their nature, require from half an hour to an hour's time, remains still to be proved by actual experiment."

